# The Action of Poison Gases

**Published:** 1919-08-23

**Authors:** 


### THE NEW DERMATOLOGY.

### IV. The Action of Poison Gases.

The last article of this series dealt in a general way with the action of certain irritants on the skin, and it is now proposed to consider in detail the dermatitis produced by "mustard-gas," since it presents peculiar features of great interest.

Di-chlor-ethyl-sulphide, popularly known as "mustard-gas" and aptly christened "Yperite" by the French, was first employed by the Germans at Ypres in the summer of 1917, and was discharged in shells marked with a yellow cross—hence the name "yellow-cross gas." It is a dark brown, oily-looking liquid, which vapourises rapidly on exposure to the air, and it possesses a peculiarly nauseous, penetrating smell, never forgotten when once experienced, which was likened by our men to that of mustard, of garlic, of rotten apples, and even of dead bodies. Whereas phosgene and chlorine belong to the group of *pulmonary irritants*, Yperite may be classed as a *vesicant*, its value in warfare depending on its irritant effect upon the skin and mucous membranes. Phosgene is, of course, far more deadly than mustard-gas, and it kills by producing an acute œdema of the lungs themselves, whereby the alveoli are filled with fluid, the circulation through the lungs is impeded owing to pressure of the fluid on the capillaries and to thrombosis occurring in the smaller vessels of the lungs, and the blood becomes abnormally concentrated by the loss of serum. Mustard-gas, on the other hand, does not kill directly, but, by causing inflammatory necrosis of the mucous membrane of the trachea and bronchi, it predisposes to a secondary infective bronchitis and broncho-pneumonia to which the victim may succumb, although death among mustard-gas cases was a rare event. The effectiveness of this substance as a weapon of war depends on the fact that its irritant effects are not apparent until after a definite latent period, and that the vapour clings to the clothing and kit of the men exposed, so that its insidious action may be prolonged for days after exposure; by causing temporary blindness from conjunctival inflammation, and an extremely painful dermatitis; it was certainly most effective in putting a large number of men temporarily out of action, and, owing to the liability to secondary bronchitis and to various functional symptoms in these cases, convalescence was often prolonged.

### The Effect of Mustard-Gas.

The action of mustard-gas on the skin is one of its most interesting features, and the cases of dermatitis caused by it can roughly be divided into two groups; first, those in which some part of the skin has been exposed either directly to the liquid, as by splashes from a bursting shell, or to the vapour in strong concentration, as when the clothing is directly saturated with the liquid from sitting on ground on which a shell had recently exploded; and, secondly, those in which the skin of the whole body had been exposed to the vapour in relatively low concentration by sojourn in a shelled area. In the first group of cases, burns of varying severity resulted, and the character of these will be described later; in the second group, a dermatitis of striking appearance and peculiar distribution occurred. It will be obvious that some men sustained direct "burns," while exhibiting also the diffused generalised form of dermatitis.

In this latter form the appearances resemble very closely those of chrysarobin dermatitis, and the areas of skin that are most susceptible are the same in the two cases. Both chrysarobin and mustard-gas produce an acute erythema, and the distribution of this erythema will be observed to correspond to those situations in which the sebaceous glands are most abundant. In fact, the distribution is the same as that of an acute generalised seborrhœic eczema.

### The Local Pathology.

Now, according to Unna and Golodetz, the intensity of the reaction of the skin to chrysarobin depends on its relative content of oleic acid: in situations where this substance is present in abundance, *i.e.*, where the sebaceous glands are numerous, chrysarobin rapidly produces an erythematous reaction, and quickly causes the disappearance of patches of seborrhœic eczema and psoriasis; whereas in situations such as the elbows and anterior surfaces of the knees, where the sebaceous glands are scanty, the irritant effect of the drug is far less evident, and its therapeutic action correspondingly less rapid. Whether this view concerning the interaction between chrysarobin and oleic acid be true or not, the fact remains that chrysarobin and mustard-gas produce their irritant effect most evidently in the parts of the skin where the secretion of sebum is greatest, and this is to be accounted for, partly, at any rate, by the fact that both these substances are insoluble in water, but freely soluble in animal and vegetable fat. When cases of mustard-gas dermatitis were first observed it was thought that the selective action of the irritant on certain areas, such as the joint-flexures, face, and genitals, depended on the greater secretion of sweat in these parts, but the writer pointed out that it was the quantity of sebum rather than sweat that determined the sites of election, and that in these cases the palms of the hands were always spared because, though they secrete sweat in abundance, they possess no sebaceous glands whatever.

Apart from the similarity in appearance and distribution of the dermatitis produced by chrysarobin and mustard-gas, both these substances cause a purplish-brown staining of the horny layers of the skin. In both cases this discolouration follows the

☛ Previous articles appeared on May 17, June 7, and July 12, page 377.

**The New Dermatology**—(*continued*).

initial erythema, that is to say, it is late in appearance, and it does not disappear until the stained cuticle has desquamated. It has been authoritatively stated that mustard-gas may cause this staining without provoking an initial erythema, but this is probably not true, for the erythema, if slight and evanescent, often passed unnoticed.

We thus see that chrysarobin and mustard-gas possess several features in common; they are both insoluble in water while freely soluble in fats; they both cause intense irritation of the mucous membranes, *e.g.*, the conjunctivæ; both provoke a dermatitis, the intensity of which depends not only on the concentration of the irritant, but on the situation to which it is applied; and both cause a peculiar staining of the horny layers of the skin.

### Experiments in France.

Some experiments, carried out in France, on the action of mustard-gas on the skin are not without interest. The first was undertaken in order to demonstrate the relative susceptibility of certain areas of skin, and for this purpose two pieces of doubly-folded lint were applied, one to the centre of the forearm, the other to the antecubital fossa; on each piece of lint a small quantity of the liquid "gas" (insufficient to soak through the lint) was placed, and the skin of the forearm was exposed to its action for nearly twice as long as that of the antecubital space. Nevertheless, the erythematous reaction appeared sooner and was much more intense in the latter situation, thus confirming the clinical observation concerning the susceptibility of the joint-flexures.

Another experiment was undertaken with a view to testing the possible protective action of certain ointments against the gas, including one, a sample of which was taken from a German prisoner. It was shown that ointments with a fatty basis intensify the cutaneous reaction, whereas vaseline affords a certain degree of protection; no doubt the ready solubility of mustard-gas in fatty media aided its absorption by the skin.

One experiment may now be described in detail in order to illustrate this point, and also the phenomenon of what may be described as local anaphylaxis. The antecubital fossa was smeared with lanoline, and exposed to the "gas" in the manner already described for about twenty minutes. An acute erythema very rapidly appeared, and on the erythematous base blisters quickly formed such as were seen in men who had been exposed to the "gas" in strong concentration. Within twenty-four hours a burn resulted—a raw weeping, and intensely painful surface being exposed, surrounded by a wide area of œdema. The neighbouring lymphatics became engorged, and at this stage the appearances resembled very closely those seen in an *x*-ray burn. While the inflammatory process was at its height an erythematous patch, surmounted by a few superficial blisters, appeared on a portion of the skin of the opposite forearm, *which had been exposed to the "gas" a fortnight previously, but which in the meantime had completely healed.* The only explanation apparently of this phenomenon is that this portion of skin had been sensitised to the action of the irritant, and consequently reacted again when some of the poison was absorbed into the circulation from the lymphatics surrounding the severe burn on the opposite arm.

To sum up, we may say that the irritant effect of mustard-gas on the skin is dependent on the relative amount of fatty material present, and that therefore the distribution of the resulting dermatitis follows closely that of the sebaceous glands, the areas of skin in which these glands are few in number, or altogether absent, being more or less immune to the action of the "gas." And, as might be expected, persons belonging to the seborrhœic type, *i.e.*, those in whom the sebaceous glands were abnormally large and who suffered from seborrhœa, showed a greater susceptibility to mustard-gas dermatitis than others.

